# Adrenal hemorrhage: diagnostics, management, and treatment. Review and clinical update

**DOI:** 10.20452/wiitm.2025.17981

**Published:** 2025-09-26

**Authors:** Siavash Świeczkowski ‑Feiz, Sadegh Toutounchi, Ewa Krajewska, Krzysztof Celejewski, Remigiusz Gelo, Piotr Kaszczewski, Wawrzyniec Jakuczun, Urszula Ambroziak, Zbigniew Gałązka

**Affiliations:** Department of General, Endocrine, and Vascular Surgery, University Clinical Center of the Medical University of Warsaw, Warszawa, Poland; Second Department of Anesthesiology and Intensive Care, University Clinical Center of the Medical University of Warsaw, Warszawa, Poland; Department of Internal Medicine and Endocrinology Medical University of Warsaw Warszawa Poland

**Keywords:** adrenal hemorrhage, adrenocortical carcinoma, laparoscopic adrenalectomy, open adrenalectomy, pheochromocytoma

## Abstract

**INTRODUCTION:**

Adrenal hemorrhage (AH) is a rare and often underdiagnosed condition that can present with nonspecific symptoms and may be life-threatening. Accurate diagnosis and tailored management are essential.

**AIM:**

The aim of this paper was to review the literature on AH with emphasis on etiology, diagnostic approaches, management strategies, and methodological quality of available studies.

**MATERIALS AND METHODS:**

A structured search of the literature was performed. Forty-one relevant articles were included in the review. Risk of bias was assessed in 3 eligible studies (2 single-center series and 1 case series with literature review) using the Joanna Briggs Institute tools and adapted criteria.

**RESULT:**

Trauma accounted for the majority of AH cases. Nontraumatic etiologies included anticoagulation, infection, stress, and adrenal tumors, such as pheochromocytoma, adrenocortical carcinoma, and metastases. Computed tomography and magnetic resonance imaging were the key diagnostic modalities. Management strategies ranged from conservative observation and embolization to laparoscopic or open adrenalectomy, guided by hemodynamic stability, capsule integrity, and suspicion of malignancy. All assessed studies had moderate risk of bias due to retrospective design and limited sample size.

**CONCLUSION:**

AH requires high clinical suspicion and structured imaging / endocrine evaluation. Open adrenalectomy is recommended in unstable patients, in the cases of capsule rupture, or when malignancy is suspected. In patients with hemorrhage confined to the adrenal capsule, laparoscopic adrenalectomy represents the preferred surgical approach. Larger prospective multicenter studies are warranted to establish standardized guidelines.

## INTRODUCTION 

Hemorrhage is defined as an acute loss of blood from a damaged blood vessel due to traumatic or nontraumatic causes.^1^ Adrenal hemorrhage (AH) remains a diagnostic challenge because of its nonspecific clinical presentation.^2^;^3^ It is a rare but potentially life-threatening condition that can lead to acute adrenal insufficiency and may be fatal. Reported causes include septicemia, coagulopathy, bleeding diathesis, trauma, and adrenal tumors.^2^;^3^;^4^ Based on autopsy data, AH is estimated to occur in 0.14% to 1.1% of deaths. The pediatric population carries a 7-fold higher risk of mortality from AH. In particular, Waterhouse–Friderichsen syndrome, usually associated with severe *Neisseria meningitidis* infection, is linked to 55%–60% mortality.^3^;^5^;^7^

Clinical presentation of AH is heterogeneous and nonspecific. Symptoms depend on the severity of bleeding, the size of the hematoma, and the extent of adrenal tissue damage. Patients may present with abdominal pain, back pain (often misinterpreted as radicular pain), nausea, vomiting, hypotension, confusion, fever, or a hemoglobin (Hb) decrease by 1.5 g/dl or greater.^3^;^8^ Although AH was historically diagnosed most often postmortem, the increasing availability of computed tomography (CT) and magnetic resonance imaging (MRI) has led to more frequent incidental detection.^3^;^9^ Management strategies remain unclear and largely depend on the patient’s clinical condition and the experience of the treating endocrinologist or surgeon. Hemodynamically unstable patients may require intensive medical treatment, including blood transfusion, management of adrenal insufficiency, and surgical intervention, either laparoscopic or open.

**Table 1:** Risk of bias assessment in the included studies

Author	Study type	Participant selection	Method (randomization / blinding)	Data reporting	Sample size	Overall risk of bias
^3^	Single‑clinic experience	Limited to 1 center	No randomization or blinding	Mostly complete	Small own cohort	Moderate**^a^**
^4^	Single‑center experience	Limited to 1 center	No randomization or blinding	Mostly complete	Small own cohort	Moderate**^a^**
^8^	Case series + review (6 original cases + 133 cases from the literature)	Diverse sources, heterogeneous	No randomization	Inconsistent reporting	Small own cohort, large literature sample	Moderate**^a^**

**a **Risk of bias assessment was conducted for 3 clinical studies and all were classified as having a moderate risk of bias, which means that their findings are valuable and can be used to support clinical conclusions.

**Table 2:** Risk factors for adrenal hemorrhage**^a^**

Conditions predisposing to adrenal hemorrhage		Examples
Trauma (80% of the cases)		-
Stress		Burns Hypotension Surgery (particularly orthopedic surgery)
Infectious disease		Sepsis caused by *Neisseria meningitidis*, *Pseudomonas aeruginosa, Escherichia coli, Bacteroides fragilis, Streptococcus pneumoniae*
Medication		Anticoagulants Antiplatelets Nonsteroidal anti-inflammatory drugs Synthetic adrenocorticotropic hormone Glucocorticosteroids
Hematologic disorders		Antiphospholipid syndrome Systemic lupus erythematosus Heparin-induced thrombocytopenia Other thrombocytopathies Thrombocytosis
Obstetric causes		Pregnancy Postpartum period Pre-eclampsia
Perinatal injury		Asphyxia Perinatal hypoxia Sepsis Fetal hematologic disorders
Adrenal gland tumor	Primary	Pheochromocytoma, adrenocortical cancer, myelolipoma, lipoma, hematoma, angioma, adenoma, pseudocyst
	Metastatic	Lung cancer, renal cancer, breast cancer, colon cancer, thyroid cancer, gallbladder cancer, melanoma
Gastrointestinal diseases		Acute pancreatitis

**a **Republished from Karwacka et al^4^ with permission under the Creative Commons CC BY-NC-ND license.

## AIM

The aim of this review was to synthesize evidence on AH in adults, in terms of etiologies / risk factors, imaging-based diagnosis, and management strategies, as well as to identify gaps for future research.

## MATERIALS AND METHODS

### Protocol and registration

No protocol was registered for this review.

### Eligibility criteria

We included primary clinical studies (randomized or observational), case series (≥5 patients), and informative case reports on AH containing data on etiology, imaging, management, or outcomes. We excluded pediatric-only cohorts, non-English papers, and nonoriginal articles without extractable data.

### Information sources

The information was extracted from PubMed and Scopus, from 1990 to 2023. We also screened reference lists and relevant guidelines.

### Search strategy

Databases were searched using strings in the following format: (“adrenal hemorrhage” OR “adrenal hemorrhage” OR “adrenal bleeding”) AND (“diagnosis adrenal hemorrhage / hemorrhage” OR “imaging adrenal hemorrhage / hemorrhage” OR “management adrenal hemorrhage / hemorrhage”).

### Selection process

Two reviewers (RG and PK)independently screened titles / abstracts and full texts. Disagreements were resolved by consensus or by a third reviewer (JW).

### Data collection process

We extracted study design, setting, sample size, etiology / risk factors, imaging features, interventions (conservative, embolization, laparoscopic / open surgery), and outcomes (mortality, adrenal insufficiency, complications).

### Data items

Primary outcomes included all-cause mortality, hemodynamic instability, and need for urgent surgery. Secondary outcomes comprised imaging characteristics across hemorrhage stages, distribution of tumor histologies, and endocrine activity.

### Risk of bias assessment

The risk of bias was evaluated using the Joanna Briggs Institute (JBI) critical appraisal tools for case series, with adapted criteria applied to case reports. For the study combining a case series with a literature review,^8^ relevant elements of the JBI tool were applied to assess methodological quality. All included studies were rated as having a moderate risk of bias, mainly due to limitations inherent to retrospective single-center designs, small sample sizes, and a lack of randomization or blinding.

### Statistical analysis

Given the heterogeneity and predominance of case series / reports, a formal meta-analysis was not performed. Descriptive statistics (counts and proportions) were used where appropriate.

### Ethics

Ethical approval was not required for this systematic review of published literature.

## RESULTS

### Study selection

From the available literature, 41 articles were selected as relevant to AH, and included in the review. Among these, narrative reviews, single case reports without clinical outcomes, and purely imaging-focused articles were excluded from the risk of bias assessment. In total, 3 studies were included for detailed methodological appraisal: 2 single-center retrospective series^3^;^4^ and 1 combined case series with literature review.^8^

### Study characteristics

Trauma accounted for the majority (ca. 80%) of AH cases reported in the literature. Nontraumatic etiologies included anticoagulation / coagulopathy, sepsis / Waterhouse–Friderichsen syndrome, stress-related states, and adrenal neoplasms (pheochromocytoma, adrenocortical carcinoma, metastases). CT and MRI were the principal diagnostic modalities, with stage-specific imaging features consistently described.^2^;^9^;^27^

### Risk of bias

The overall risk of bias in the included studies was determined as moderate, reflecting the limitations of retrospective, single-center designs, small patient numbers, and a lack of randomization or blinding.

### Results of individual studies and synthesis

Świeczkowski-Feiz et al^3^ and Karwacka et al^4^ reported clinical presentations and management outcomes of nontraumatic AH in single-center cohorts. Marti et al^8^ analyzed 6 original cases and 133 cases from the literature, providing insights into etiology, imaging characteristics, and treatment strategies Table 1. Across the studies, management ranged from conservative care and selective embolization to laparoscopic or open adrenalectomy, guided primarily by hemodynamic stability and capsule integrity.

## DISCUSSION

### Anatomic and physiologic background

The adrenal gland is supplied by the superior, middle, and inferior suprarenal arteries, branches of the inferior phrenic artery, abdominal aorta, and renal artery, respectively. This vascular configuration, known as a vascular dam, results in 50–60 small arterial branches that form a delicate subcapsular plexus around the zona glomerulosa. Consequently, the adrenal cortex is particularly susceptible to destruction during hemorrhage. Venous drainage occurs through a single adrenal vein, characterized by numerous longitudinally arranged smooth muscle cells in its wall. This unique structure makes the vein sensitive to catecholamines. Elevated catecholamine levels induce vasoconstriction, increased venous resistance, and blood stasis, predisposing the adrenal gland to ischemic necrosis of small arterioles and subsequent hemorrhage upon reperfusion. Catecholamines further promote platelet aggregation and vascular spasm, contributing to adrenal vein thrombosis under certain conditions.^10^;^11^;^12^;^32^

### Etiology and risk factors

Trauma remains the strongest risk factor, responsible for approximately 80% of the reported AH cases. The remaining 20% were attributed to nontraumatic causes, including anticoagulant therapy, hematologic disorders, infectious diseases (eg, Waterhouse–Friderichsen syndrome), obstetric complications, perinatal injury, adrenal neoplasms (pheochromocytoma, adenoid cystic carcinoma [ACC], metastases), and gastrointestinal disorders.^9^;^13^;^14^;^15^ This broad spectrum highlights the need for high clinical suspicion, especially in patients with sudden abdominal, flank, or back pain without obvious trauma Table 2.

### Diagnostic imaging

The diagnostic pathway for suspected AH includes ultrasound (US), CT, and MRI. US is often the initial modality in emergency settings, where acute hematomas appear solid and echogenic, later evolving into mixed echogenicity and eventually anechoic cystic lesions with possible calcified walls after 1–2 weeks. Doppler US demonstrates the avascular nature of hematomas.^16^;^17^;^28^

CT remains the most widely used modality for confirming AH. Nontraumatic hemorrhage typically appears round or oval, with periadrenal extension if the capsule ruptures. Acute and subacute hematomas have radiodensity of 50–90 Hounsfield units, later evolving into organized pseudocysts with hypoattenuating centers and calcifications. In most cases, conservative management leads to complete resolution within 1 year. Calcifications appear in the early stages of hemorrhage development.^16^;^17^;^28^

MRI provides the most detailed staging of AH based on Hb degradation products. In the acute stage (<⁠7 days), intracellular deoxy-Hb yields isointense to hypointense T1 signals and hypointense T2 signals.^10^;^16^;^29^ During the early subacute phase (2–7 days), intracellular met-Hb produces hyperintense T1 and hypointense T2 signals, initially at the periphery of the hematoma. By the late subacute phase (7–14 days), extracellular met-Hb is hyperintense on both T1 and T2 images.^10^;^16^;^28^;^29^ In chronic hematomas, hemosiderin deposition and fibrous capsule formation cause a hypointense rim on both sequences.^10^;^16^;^28^;^29^ A characteristic “train-track appearance” has been described in early nontraumatic AH, where the periphery enhances while the central portion shows low attenuation, resembling parallel railroad tracks.^17^ Recognition of this early sign may facilitate prompt diagnosis before hematoma formation is complete.

### Histology and pathology

Histologic evaluation confirms that pheochromocytomas are the most frequent hemorrhagic adrenal tumors, followed by ACCs and metastatic lesions. In a series by ^3^ 28.2% of hemorrhagic tumors were pheochromocytomas, 23.1% were ACCs, and 7.7% were metastases. ^4^ described 23 cases, with 9 involving surgical intervention. Histopathology confirmed adrenal hematoma, and 1 case demonstrated hormonal activity. ^8^ analyzed 6 original and 133 literature cases, reporting pheochromocytomas and ACCs as the predominant hemorrhagic tumors, including cases with hormonal activity. Importantly, AH may be the first and only manifestation of adrenal malignancy, necessitating thorough endocrine and oncologic evaluation in all cases.^3^;^4^;^9^

**Figure 1:**
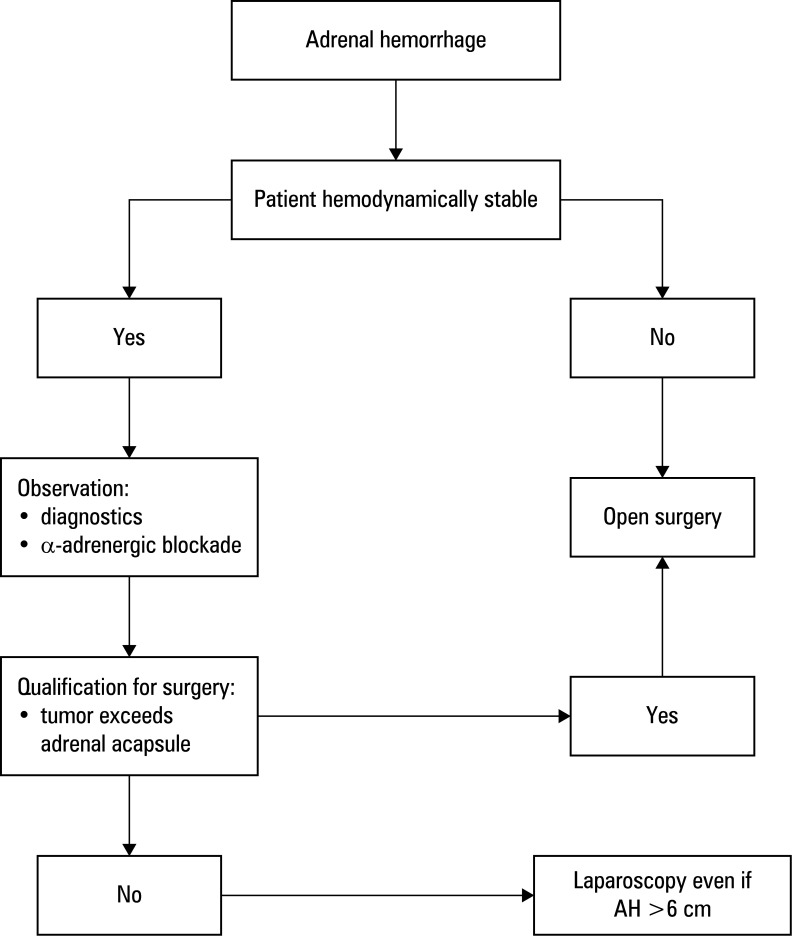
Management algorithm in adrenal hemorrhage (AH)

### Management strategies

Management depends on hemodynamic stability, capsule integrity, and suspicion of malignancy. In unstable patients or in the cases of capsular rupture, urgent open adrenalectomy is indicated to prevent catastrophic vascular or organ injury.^15^ In stable patients with contained hemorrhage, laparoscopic adrenalectomy is the gold standard, even for selected tumors exceeding 6 cm, provided surgery is performed in experienced centers.^25^;^26^;^27^ Reports indicate that large adrenal tumors (>10 cm) may also be safely removed laparoscopically without compromising oncologic outcomes Figure 1.^25^;^26^;^27^.

Endocrine assessment is mandatory in all cases. Patients should undergo screening for pheochromocytoma with plasma free or urinary fractionated metanephrines.^19^ Cortisol excess should be excluded via the 1-mg dexamethasone suppression test, with further evaluation if abnormal.^19^ Primary aldosteronism should be considered in hypertensive or hypokalemic patients by calculating the aldosterone / renin ratio.^20^ This structured approach, recommended by the Endocrine Society and European Society of Endocrinology, ensures accurate risk stratification of functional lesions.^20^;^21^;^22^;^30^

Preoperative medical optimization is critical for pheochromocytoma. Both α-adrenergic blockade with doxazosin and β-blockade (if required) reduce perioperative risk.^37^;^38^;^39^ Despite adequate preparation, intraoperative hypertensive surges or arrhythmias may still occur during anesthesia induction, pneumoperitoneum, or tumor manipulation.^37^;^38^;^39^ Failure to reach pharmacologic optimization may result in life-threatening crises. Embolization has been reported as a temporizing option in selected cases, followed by definitive surgery.^23^

### Own experience and observations

In our cohort, hemorrhagic adrenal tumors had a mean diameter of 7 cm. Hemorrhage was the first and only manifestation of underlying pathology in many cases.^3^ Histology confirmed ACC in 4 patients (1 hormonally active) and pheochromocytoma in 11 individuals, of which 5 were hormonally active. Interestingly, several pheochromocytomas were biochemically silent preoperatively, likely due to hemorrhagic necrosis masking catecholamine secretion. Operative times for laparoscopic and open adrenalectomy were consistent with published reports, with surgeon experience strongly influencing the outcomes. These findings reinforce the fact that every AH must be managed as a potential pheochromocytoma or ACC until histologically proven otherwise.^3^

### Summary statement

AH represents a diagnostic and therapeutic challenge due to its rarity, variable presentation, and diverse etiologies. Every AH occurring within a mass should be regarded as a possible pheochromocytoma or ACC until definitively excluded on histopathology.^3^;^37^ Preoperative endocrine evaluation and α-adrenergic blockade are mandatory. The choice of a surgical approach must consider tumor size, morphology, hemorrhagic dynamics, patient condition, and surgeon expertise.^25^;^41^ While laparoscopic adrenalectomy remains feasible for most contained lesions, open adrenalectomy is recommended in unstable patients and in the cases of capsule rupture with extracapsular bleeding.^25^;^41^ Ultimately, all patients with AH should be prepared for expedited surgery following comprehensive evaluation, as conservative management carries the risk of missing underlying malignancy.

## CONCLUSIONS

AH is a rare and underdiagnosed condition that should always be considered in patients presenting with nonspecific symptoms, such as sudden back, flank, or abdominal pain. Tumors, including pheochromocytomas, ACCs, and metastases carry a particularly high risk of hemorrhage. In the cases of hemorrhagic tumors with capsule rupture, laparoscopic surgery is associated with a significant risk of vessel or adjacent organ injury, and therefore open surgery is recommended. The timing of surgical intervention vs conservative observation remains debated in the literature; however, surgery should always be considered when histopathologic diagnosis is required.

Although laparoscopic adrenalectomy has become routine in surgical practice and offers well-documented advantages, such as shorter operative time, less postoperative pain, lower wound infection rates, and reduced risk of postoperative hernias in patients with hypercortisolism, these benefits apply primarily to contained lesions. In contrast, open surgery remains the preferred approach in the setting of capsule rupture or suspected malignancy.

Overall, AH management requires high clinical suspicion, structured imaging, and endocrine work-up. Treatment must be individualized according to hemodynamic stability, capsule integrity, and oncologic risk. Open surgery is indicated in unstable patients, suspected malignancy, or ruptured tumors, while laparoscopic adrenalectomy is feasible and safe in selected cases with contained hemorrhage.
